# T-Cell Immunotherapies Targeting Histocompatibility and Tumor Antigens in Hematological Malignancies

**DOI:** 10.3389/fimmu.2020.00276

**Published:** 2020-02-21

**Authors:** Valérie Janelle, Caroline Rulleau, Simon Del Testa, Cédric Carli, Jean-Sébastien Delisle

**Affiliations:** ^1^Centre de Recherche de l'Hôpital Maisonneuve-Rosemont, Montréal, QC, Canada; ^2^Département de Médecine, Université de Montréal, Montréal, QC, Canada; ^3^Division Hématologie et Oncologie, Hôpital Maisonneuve-Rosemont, Montréal, QC, Canada

**Keywords:** histocompatibility antigens, tumor-specific antigens (TSA), tumor-associated antigens (TAA), transgenic T-cell receptors, T-cell immunotherapy, viral antigens, allogeneic stem cell transplant, chimeric antigen receptor (CAR)

## Abstract

Over the last decades, T-cell immunotherapy has revealed itself as a powerful, and often curative, strategy to treat blood cancers. In hematopoietic cell transplantation, most of the so-called graft-vs.-leukemia (GVL) effect hinges on the recognition of histocompatibility antigens that reflect immunologically relevant genetic variants between donors and recipients. Whether other variants acquired during the neoplastic transformation, or the aberrant expression of gene products can yield antigenic targets of similar relevance as the minor histocompatibility antigens is actively being pursued. Modern genomics and proteomics have enabled the high throughput identification of candidate antigens for immunotherapy in both autologous and allogeneic settings. As such, these major histocompatibility complex-associated tumor-specific (TSA) and tumor-associated antigens (TAA) can allow for the targeting of multiple blood neoplasms, which is a limitation for other immunotherapeutic approaches, such as chimeric antigen receptor (CAR)-modified T cells. We review the current strategies taken to translate these discoveries into T-cell therapies and propose how these could be introduced in clinical practice. Specifically, we discuss the criteria that are used to select the antigens with the greatest therapeutic value and we review the various T-cell manufacturing approaches in place to either expand antigen-specific T cells from the native repertoire or genetically engineer T cells with minor histocompatibility antigen or TSA/TAA-specific recombinant T-cell receptors. Finally, we elaborate on the current and future incorporation of these therapeutic T-cell products into the treatment of hematological malignancies.

## Introduction

**Figure 1:**
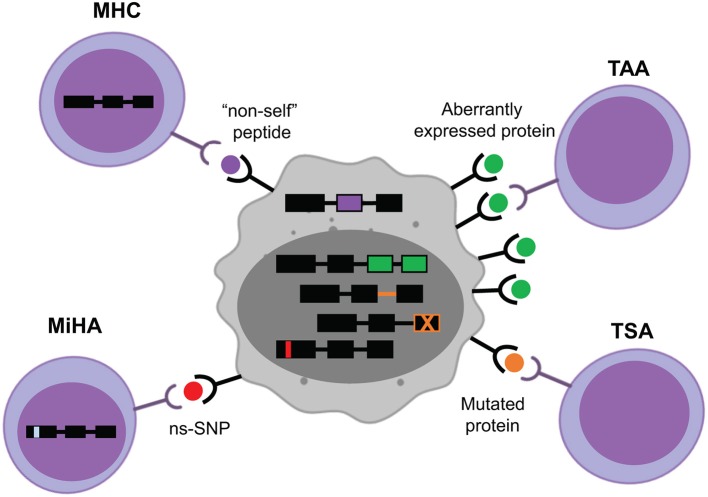
Target MHC-associated antigens in hematological cancers. Major histocompatibility complex (MHC)-associated antigens may originate from viral components, such as the episomal translation of Epstein-Barr Virus proteins (purple). The majority of known minor histocompatibility antigens (MiHA) are generated by non-synonymous single nucleotide polymorphisms (ns-SNP) between the donor and the recipient of the T-cell therapy (red). Tumor-specific antigens (TSA) arise from intronic or exonic mutations unique to the tumor cells (orange). Tumor-associated antigens (TAA) come from aberrantly expressed proteins in cancer cells (green).

Allogeneic hematopoietic cell transplantation (AHCT) remains to this day the most widely used form of cancer cellular immunotherapy. Several studies in both humans and animals have conclusively shown that the recognition of alloantigens by T cells is central to the so-called “graft-vs.-tumor” (GVT) that occurs following AHCT (1–3). However, the recognition by donor T cells of major and minor histocompatibility antigens (MiHA), encoded by germline polymorphisms and expressed on malignant and normal host hematological cells as well as on non-hematological cells, can also result in graft-vs.-host disease (GVHD) (4). Despite several decades of research, the potentially lethal GVHD reactions are still the major limitation to the use of alloreactivity to treat blood cancers with AHCT. Recent antigen identification and characterization methods, coupled with refined cell manipulations and cell transfer procedures, may allow for an effective separation of the GVT and GVHD effects when targeting alloantigens. Moreover, other antigens are inspiring immunotherapeutic strategies that can be implemented in AHCT and non-transplant settings (5). The tumor-specific antigens (TSA) refer to major histocompatibility complex (MHC) class I or II-associated peptides that are found solely at the surface of tumor cells. Often resulting from acquired genetic variants, these antigens can stimulate vigorous T-cell responses and will be extensively described below. T-cell immunotherapies targeting unmutated MHC-associated antigens, including viral antigens and tumor-associated antigens (TAA) will also be described in the context of blood cancers. This review will focus on the current status of immunotherapeutic approaches, particularly those exploiting genetic variants, native and acquired, for the treatment of hematological malignancies. These antigens are almost exclusively relevant to the context of immune therapies using conventional T cells, CD8^+^ and CD4^+^, that recognize their MHC-bound peptide antigen through a T-cell receptor (TCR) composed of an alpha and beta chain (Figure 1). T-cell therapies targeting non-polymorphic antigens and the use of other immune cell types will also be briefly discussed and put in context of the current status of cellular immunotherapies for blood neoplasms. The implementation of T-cell therapies targeting relevant antigens for hematological cancers hinges on a detailed knowledge of the targets, T-cell biology, gene engineering, *ex vivo* cell processing methods and clinical expertise. As such, these therapies represent a formidable challenge but also an opportunity to make paradigmatic advances in blood cancer treatment and oncology in general.

## Target Antigens in Hematological Cancers

### Histocompatibility Antigens, Majors, and Minors

AHCT's curative potential relies substantially on the GVT effect, which is largely based on the recognition of histocompatibility antigens by allogeneic T cells. These antigens result from the translation of germline-encoded genetic variants (6–10). However, standard AHCT is a personalized but markedly unspecific form of immunotherapy. The broad repertoire of allogeneic T cells transferred with the graft react against a multitude of host derived antigens. These can be expressed on several cell and tissue types, inducing GVHD in most recipients despite prophylactic immunosuppression (11, 12). Thus, the curative potential of AHCT relies on the transfer of histo-incompatible T cells recognizing germline genetic variants on neoplastic cells (13–17). Histocompatibility antigens are prime targets for T cells because they stimulate a high avidity T-cell repertoire. Histocompatibility antigens are not expressed in donor thymus, therefore T cells recognizing histocompatibility antigens with high functional avidity do not undergo negative selection prior their adoptive transfer in patients (18, 19). Moreover, the high frequency of GVHD occurrence in recipient of multiparous female donors hints at the possibility of sensitization to host recipient antigens and the mobilization of a memory T-cell repertoire against these antigens (20). Thus, AHCT patients receive a treatment which is targeted to a mostly unknown set of antigens by an equally elusive T-cell repertoire leading to frequent toxic “on-target/off-tumor” immune responses. The discovery and characterization of relevant transplantation antigens nonetheless hold great promise for the design of immunotherapies that could enhance the GVT effect and limit the occurrence of GVHD. The development of such immunotherapies depends on the identification of antigens that are specifically, or at least preferentially, expressed on hematopoietic and/or malignant cells (6, 21). As such, Human leukocyte antigen (HLA) (the major histocompatibility antigens) and MiHA mismatches can be harnessed to treat hematological cancer patients.

The frequency of T cells capable to target mismatched HLA molecules is very high (1–10%) (22–24). Given the likelihood of severe GVHD occurrence when AHCT is performed across HLA barriers, refinements in HLA typing in the last years have improved outcomes due to better matching (25, 26). To this day, HLA compatibility remains a key variable in AHCT and most centers consider that a related or unrelated HLA identical donor is the best donor. However, recent advances in cell handling and GVHD prophylaxis now enable the use of partially HLA mismatched cord blood and related haplo-identical donors, with results that are comparable to those obtained with HLA matched donors (27, 28). In both cases, the risk of GVHD (especially chronic GVHD) is surprising low. Although the reasons for this are incompletely understood, several factors, such as the intensity of the immunosuppression in haplo-identical AHCT, or the intrinsic features of the graft in terms of cell composition and functionality in cord blood transplants, may contribute to this observation (29, 30). Moreover, in certain circumstances, the risk of relapse appears to be lower following these mismatched transplants, arguing in favor of enhanced GVT in these settings (31–33). Based on the presumption that anti-HLA T-cell reactivity is an effective anti-neoplastic mechanism, the infusion of intentionally mismatched peripheral blood mononuclear cells following chemotherapy is being investigated as a form of immunological consolidation after chemotherapy (34). Such “microtransplantations” resulted in improved leukemia outcomes relative to the usually reported survival and leukemia-free survival rates, despite the absence of prolonged and significant engraftment (35, 36). These results need nonetheless to be confirmed and the underlying mechanisms better defined. Several questions remain about the relative contribution of CD4^+^ and CD8^+^ HLA-specific T cells and other cell types, such as natural killer (NK) cells in the recognition of HLA-mismatched cellular targets [reviewed in Paul and Lal (37)]. The infusion of HLA mismatched NK cells has led to promising clinical results, confirming a direct anti-neoplastic effect (38, 39). Hence, the respective impacts of T-cell and NK-cell reactivity in HLA mismatched transplants and other cell therapy approaches are still unknown but may account for the effects on GVT and GVHD observed in haplo-identical and cord blood transplants. To this date, no clinical studies using *ex vivo* expanded anti-HLA reactive T cells have been reported. Although this may be fraught with the risk of inducing severe GVHD, the design of anti-HLA T-cell therapy targeting the class II antigens which have a more restricted tissue expression (with high expression in blood cancer subtypes) may be considered (40, 41).

**Figure 2:**
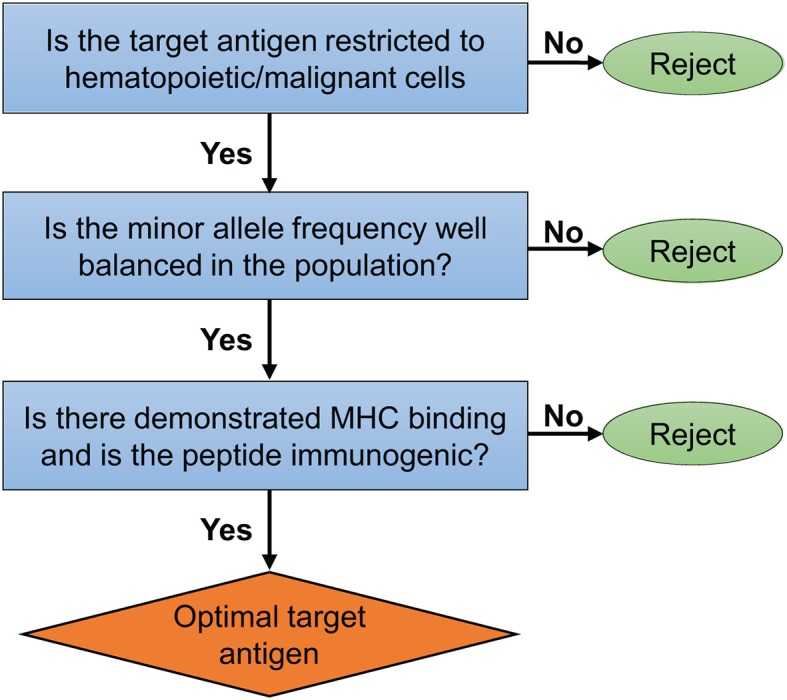
Ideal MiHA target selection. Important criteria and proposed algorithm to select optimal target MiHA for immunotherapy of blood cancers.

In the context of HLA-matched AHCT, alloreactive donor T cells (CD4^+^ and CD8^+^) recognize MHC-bound polymorphic peptides derived from the host proteome and known as the MiHA. Both MHC class I and class II molecules have been shown to present MiHA (2, 6, 42–47). Most of the molecularly characterized MiHA are encoded by autosomal genes that differ between patient and donor secondary to germline encoded non-synonymous single nucleotide polymorphisms (ns-SNP). However, the true contribution of ns-SNP to MiHA disparities is unknown. Several non-SNP events, such as alternative proteasome degradation, non-presentation of allelic variants, Y-chromosome derived peptides, polymorphic proteins created by frameshift insertions or deletions [reviewed in Griffioen et al. (48)] can also generate MiHA. However, these are more difficult to characterize using currently available methods (45, 49). Recent evidence suggest that the genetic origin of the MiHA presented by MHC class I is not random, with specific exomic regions coding for proteins being overrepresented among the repertoire of MHC-peptides directly assessed by proteomic methods (45, 50). This indicates that relying on ns-SNP detection to predict MiHA's sequences is fraught with limitations as only 0.5% of ns-SNP generate MHC-associated peptides (6, 51). More comprehensive proteogenomic analyses, including the direct identification of MHC-associated peptides by mass spectrometry appears to be required to permit the robust, and high-throughput, identification of candidate MiHA that derive from ns-SNP (6, 51). One shortcoming of current methods to define MHC-associated peptides as candidate antigens, is that our current proteomics and bioinformatics tools are better at identifying MHC class I than class II-associated peptides (52, 53). In addition to the confirmation of presentation by the MHC, candidate MiHA for immunotherapy should fulfill several other criteria (Figure 2). Perhaps the most important is tissue restriction. In the context of AHCT, the expression of the source protein of a given MiHA should be restricted to the hematopoietic system and the malignant cells. Determining tissue distribution can be achieved through several methods including bioinformatics mining of tissue gene expression atlases to standard biochemical and histological methods (6). Moreover, a practical consideration is that the minor allele frequency (MAF) of a MiHA sequence should be well-balanced in the population to enhance the odds that there will be a mismatch between the donor and recipient. Pre-clinical studies in mice demonstrated the curative potential (without causing GVHD) of injected T cells primed against a single MiHA (54–56) offering solid proof of principle for the development of MiHA-based immunotherapeutics in humans.

### Tumor-Specific Antigens (TSA)

The genetic mutations that characterize the neoplastic process can result in acquired ns-SNP with altered reading frames and the translation of proteins with different amino acid sequences (57). Once degraded and presented by MHC molecules at the cell surface, these altered sequences can be recognized by the host T cells. Since the mutations giving rise to these so-called neoepitopes are present only in cancer cells, the resulting antigens are deemed specific for the tumor. TSA are therefore thought to be most prevalent in highly mutated cancers, such as melanoma and lung cancer. The correlation (albeit very imperfect) between mutation load and responses rates to immune checkpoint (CTLA-4 and PD-1) blockade reinforces the notion that the mutanome is immunologically relevant (6, 58–60). The discovery of TSA has provided new hopes for the field of cancer vaccines with several trials launched in the last decade (61–66). It has also provided a rationale to explain the success of tumor infiltrating lymphocytes (TIL) infusions in certain cancers (67). The identification of putative patient-specific tumor antigens generated by somatic mutation is unfortunately insufficient as most mutations identified in tumor-expressed genes do not generate neoepitopes capable of stimulating T-cell responses. It has been estimated that only 10% of the non-synonymous mutations in tumor cells can generate mutant peptides with high MHC affinity (68), while only 1% of peptides with high MHC affinity can be recognized by T cells in patients (69). Moreover, a large fraction of these mutations are not shared between patients and often not by all cells comprised in the tumor or metastases (70, 71). Such heterogeneity forces the development of highly personalized approaches for immunotherapy. Unlike many solid tumors, hematologic cancers usually carry a low mutation burden and consequently, TSA are predicted to be much less frequent in these neoplasms (72). However, specific B-cell receptor sequence in B-cell malignancies (idiotype) offer an opportunity to specifically target mature B-cell cancers (73). Likewise, the presence of well-characterized fusion proteins in leukemia, notably the BCR-ABL fusion in chronic myelogenous leukemia and acute lymphoid leukemia, enabled the demonstration that circulating T cells could recognize neo-epitopes created by the fusion (74). The infusion of T cells targeting BCR-ABL fusion epitopes in three acute lymphoid leukemia patients bearing the fusion has been associated with molecular remission and trafficking of the antigen-specific T cells to the bone marrow, hence providing a rationale to pursue the development of TSA-based immunotherapy in blood cancers (75).

It is now increasingly recognized that the acquired genetic variants only represent a fraction of the aberrancies leading to an altered MHC-ligandome on cancer cells. Recent evidence shows that transcription and translation of presumed non-coding genetic regions may significantly alter the immunogenicity of malignant cells. These antigens are different from the well-known TAA which originate from canonical reading frames that are either overexpressed and/or abnormally expressed in neoplastic cells (see below). Therefore, a subclassification for TSA has recently been proposed; mutated TSA (mTSA) and aberrantly expressed TSA (aeTSA) (76). The mTSA derive from mutated DNA sequences in canonical genes that can be either exonic or non-exonic (77, 78) and the aeTSA arise from aberrant and cancer-specific expression of unmutated non-canonical transcripts that are not expressed in normal tissues, including thymic medullary cells (mTECs), which has crucial importance for central tolerance. The aeTSA combine the immunological characteristics of MiHA and mTSA, despite being non-polymorphic and shared between individuals and cancer cells like TAA (76, 79). Importantly, aeTSA that derive from unmutated non-exonic sequences (introns, intergenic regions, etc.) may be very abundant, as revealed by proteogenomic methods in human acute lymphoblastic leukemias and lung cancer samples (76, 80).

### Tumor-Associated Antigens (TAA)

Neoplastic cells can overexpress, or aberrantly express, unmutated proteins that are recognized by the immune system (46, 81–83). At present, several TAA have been identified across many cancer types. They are categorized traditionally into four groups: antigens encoded by cancer-gonads genes, embryonic/differentiation genes, overexpressed antigens, and viral antigens. The inclusion of viral antigens as TAA is problematic for several reasons, the most important being that virus-derived antigens are non-self and do not contribute to central tolerance in the thymus like the other TAA. Virus-specific T cells have high functional avidity and have repeatedly been shown to be highly effective for the treatment of Epstein-Barr virus (EBV)-associated lymphoma, particularly in the post-transplant setting (84). Despite issues related to central tolerance, TAA can elicit T-cell responses and TAA-specific T cells can be found at high frequency in the circulating T-cell repertoire of normal individuals (79, 85). Several immunotherapies have been devised to target TAA derived from proteins, such as WT1, NY-ESO-1, PRAME, Proteinase 3, MAGE-A3 in blood cancers and despite inherent limitations, TAA have practical advantages for the design of immunotherapies. The most evident being that being non-polymorphic, they are applicable to a large number of patients and can be prepared using standardized reagents.

### MiHA, TSA, TAA, Which Targets to Choose for Blood Cancer Immunotherapy?

The ideal antigenic targets should be highly cancer-specific, be universally applicable to all patients and cancer types and enable treatment without the requirement for AHCT. This last decade has seen the rise of anti-CD19 chimeric antigen receptor (CAR)-modified T cells which fulfill some of these characteristics (86). Despite excellent clinical results in childhood acute lymphoblastic leukemia (ALL), diffuse large B-cell lymphoma (DLBCL) and myeloma, current CAR-based approaches are limited to a subset of B-cell antigens [CD19, CD22 (87), B-cell maturation antigen—BCMA (88)]. This is partly because the on-target/off-tumor reactivity leading to normal B-cell depletion is easy to palliate with exogenous gammaglobulins. Targeting other cells, notably those of the myeloid lineage with CAR therapy may prove to be more difficult as the most promising antigens are also expressed by normal progenitor cells. Finally, toxicities related to this therapy are substantial (89). In fact, the cytokine release syndrome (CRS) and neurological toxicities that follow CAR T-cell infusions require careful patient follow up. The CRS involves fever, hypotension and hypoxia that can rapidly degenerate into organ dysfunction if not treated with anti-cytokine therapy. Likewise, seemingly mild cognitive deficits can rapidly degenerate into encephalopathy and seizure if left untreated. Hence, it is likely that pursuing MHC-associated antigens originating from genetic variations, or variations in the expression of unmutated genetic sequences will offer the promise of immunotherapy for the effective and safe treatment of the full spectrum of blood cancers.

Because they are encoded by germline polymorphisms instead of somatic mutations, MiHA possess features that make them attractive for immunotherapy (6). In contrast to TSA, suitable MiHA are more likely to be expressed by all neoplastic cells and applicable to a large number of patients (71, 90, 91). However, this limitation may not be as important if TSA are derived from shared driver mutations or fusion proteins. For both MiHA and TSA, the use of high avidity T-cell repertoires remains a most appealing element. However, devising and implementing immunotherapies targeting shared epitopes is more convenient. This is a major aspect driving TAA-specific strategies. Lastly, aeTSA may be shared by many tumors, while being non-polymorphic and not inducing central immune tolerance. These characteristics would make aeTSA ideal targets, but much more work is needed in order to evaluate the therapeutic potential of these antigens in humans. The next section will review current and future T-cell therapy strategies (both autologous, and donor-derived in the context of AHCT) directed against these various antigen types.

## T-Cell Immunotherapy Strategies

The development of methods to identify and characterize MHC-associated antigens resulting from genetic variants is motivated by a strong impetus to design T-cell therapies to treat neoplastic diseases. These T-cell therapies may be used alone or in combination with other approaches, such as vaccination and immune checkpoint blockade but this review focuses on the current status of T-cell therapies aimed at MHC-associated peptides to treat hematological cancers.

T-cell therapies can be antigen agnostic (administered without precise knowledge of the antigens targeted), such as in unmanipulated donor lymphocyte infusions (DLI) and TIL infusion, or targeted to known antigens. The administration of antigen-specific T cells requires prior *ex vivo* manipulations for enrichment and/or expansion of T cells bearing native TCR specific to the targeted antigens. Alternatively, genetic engineering can enable the production of large numbers of TCR transgenic T cells directed against a given antigen.

### Antigen Agnostic Approaches

The use of DLI has been one of the most conclusive proof of the GVT effect in AHCT (i.e., objective responses following the infusion of donor cells without other treatment). However, the efficacy of donor T cells, collected after AHCT and infused in graded doses, has yielded variable results and has a risk of triggering GVHD in 60–70% of patients (92). There is substantial variability in the response rates to DLI based on the underlying disease [from close to 100% in chronic phase chronic myelogenous leukemia to 15–40% in acute leukemia (93)], the disease burden, the timing of administration (pre-emptive vs. advanced disease) and the use of concomitant treatments. Most of the experience in DLI was gained in HLA-matched transplant settings, where MiHA mismatches are the drivers of the alloresponses. With no prior knowledge of the number of antigen mismatches, the tissues in which these MiHA source proteins are expressed and the number of MiHA-specific T cells present in the DLI, this form of immunotherapy does not fully harness the potential of MiHA based immunotherapy in AHCT patients. However, it has the advantage of requiring minimal manipulation and thus be rapidly accessible to a large number of AHCT patients. Other antigen agnostic T-cell therapies have been explored as treatment for solid tumors and blood cancers. An interesting approach is to attempt to exploit T cells harvested from the disease site and reinfuse them after *ex vivo* expansion. TIL therapy was pioneered in solid tumors, in melanoma particularly, where it has yielded high response rates and durable complete remissions (94). This approach is based on the assumption that T-cell populations contained in tumor beds may comprise a high frequency of tumor-reactive cells (95). This principle may also apply for several hematological malignancies. The bone marrow is a natural reservoir of antigen-experienced memory T cells and the site of disease of many blood cancers (96). As such, it may contain a large repertoire of T cells capable of recognizing the malignant hematopoietic cells. Moreover, a practical advantage is that the bone marrow is easily accessible for collection of T cells that can later be expanded *ex vivo*. Expanded autologous “MIL” (marrow infiltrating lymphocytes) from multiple myeloma patients using anti-CD3/CD28 stimulation and IL-2 revealed that the bone marrow contained a high number of myeloma reactive T cells (relative to blood derived T cells from the same patients) capable of targeting both mature and precursor myeloma cells *in vitro* (97). A clinical trial performed in 25 patients confirmed the feasibility of performing “MIL” therapy following autologous stem cell transplant in myeloma patients. The absence of a control group precludes a rigorous assessment of disease response against the standard treatment of this disease, but the authors were able to correlate the presence of anti-myeloma activity in the expanded MILs product, as well as the persistence of anti-myeloma reactivity 1 year after infusion, with favorable outcome (98).

The transfer of a large T-cell repertoire has advantages, such as broad applicability as well as the likelihood of targeting several antigens at the same time. However, antigen agnostic methods can miss the relevant targets by expanding/transferring T cells that are not specific for cancer associated/specific antigens (99). In the setting of AHCT and DLI, this can also lead to toxicity in the form of GVHD. The molecular characterization of MiHA, viral antigens, TAA and TSA now permits the development of more precise and possibly more potent T-cell therapies. This, coupled with more widely accessible T-cell manufacturing methods, allows for the use of manipulated T cells targeting MHC-associated targets in blood cancers.

### MHC-Associated Antigen-Specific Approaches in T-Cell Therapy

The current experience using T-cell therapies against MiHA, viral antigens, TSA and TAA demonstrates the possibility to expand antigen-reactive T cells in high numbers to treat patients. However, T-cell manufacturing continues to be challenging and the optimal approach to integrate these therapies in the patients' treatment trajectory remains to be determined. This section reviews the current approaches aiming to treat hematological malignancies through the specific targeting of MHC-associated antigens. A summary of the molecularly defined HLA-associated antigens that have been targeted in adoptive T-cell immunotherapy clinical studies is included in Table 1 (75, 100–107).

**Table 1:** MHC-associated antigens targeted in T-cell therapy trials for blood cancers.

**Target antigen**	**Unique or multiple antigen(s)**	**Antigen type**	**Natural vs. transgenic TCR**	**Cancer type**	**HLA restriction**	**References**
HA-1	Unique antigen	MiHA	Natural	AML, CML, ALL	A0201	(100)
P2RX7_265−273_	Unique antigen	MiHA	Natural	ALL	A2902	(101)
DPH1_334−343_	Unique antigen	MiHA	Natural	MDS	B5701	(101)
DDX37	Unique antigen	MiHA	Natural	ALL	B2705	(101)
BCR-ABL fusion	Antigen library	TSA	Natural	ALL	ND	(75)
WT-1_126−134_	Unique antigen	TAA	Natural/Transgenic	AML, ALL, MDS	A0201	(102, 103)
WT-1_235−243_	Unique antigen	TAA	Transgenic	AML, MDS	A2402	(104)
MAGE-A3	Unique antigen	TAA	Transgenic	MM	A01	(105)
NY-ESO-1/LAGE-1	Unique antigen	TAA	Transgenic	MM	A0201	(106)
LMP1, LMP2	Antigen library	Viral Ag	Natural	Lymphoma	ND	(107)

*ALL, Acute lymphoblastic leukemia; AML, Acute myeloid leukemia; CML, Chronic myelogenous leukemia; MDS, myelodysplastic syndrome; MM, Multiple myeloma. ND, not defined*.

#### MiHA

As described above, the MiHA have several conceptual advantages for immunotherapy. Vaccination against MiHA in the context of post-AHCT DLI has been reported to induce detectable MiHA-specific responses in myeloma patients. Although clinical responses were modest (transient regression or stable disease), the vaccination protocol was well-tolerated (108, 109). An alternative approach could be to vaccinate the donors prior to graft collection in order to generate a robust anti-MiHA memory T-cell repertoire in these healthy individuals, as previously done in animal models (55, 110). Unfortunately, this is difficult to envisage for several reasons, including the consequences of allosensitization in donors who may eventually require tissue, cell or organ transplantations themselves.

Cell therapy is the other approach to selectively or preferentially target MiHA. The first trial reporting on a MiHA-specific T-cell therapy strategy used MiHA-specific CD8^+^ clones obtained by co-culturing donor T cells with host-derived lymphoblastoid cells (EBV-transformed B-cells) (101). After ruling out reactivity to EBV antigens and host fibroblasts (surrogate for non-hematopoietic tissues), reactive T cells were infused. Thus, although highly specific, this approach did not rely on *a priori* knowledge of the targeted MiHA and their tissue distribution. The administration of these T-cell clones led to objective responses in 5/7 refractory relapsing leukemia patients post-AHCT. These responses were short-lived, with evidence of gradual decrease in antigen expression at disease recurrence in at least one patient, hinting at a plausible immune escape mechanism. A surprising complication was the occurrence of pulmonary toxicity, which is not seen following regular DLI. Although, the MiHA source protein could be detected in the lung tissue in one case, the patients had also received a conditioning regimen and post-infusion IL-2, which can be associated with pulmonary complications (111). Nonetheless, these findings are an additional argument to select MiHA with restricted expression to the hematopoietic system. Another trial used donor-derived T-cell lines stimulated *ex vivo* with dendritic cells loaded with the blood lineage and HLA-A0201 restricted MiHA HA-1 (100). Following up to 5 rounds of weekly stimulation with antigen loaded dendritic cells in the presence of IL-2, donor derived T-cell lines containing from 11 to 243 × 10^6^ HA-1 specific CD8^+^ T cells were infused to 3 relapsing patients post-AHCT. Although clearly demonstrating the feasibility of the approach and its innocuity (no notable GVHD), the procedure was not associated with clinical responses.

In both cases, the advanced disease status of the patients and the prolonged period of T cells in culture can be suspected as limiting factors. It was shown that repeated stimulation with antigen-loaded dendritic cells has a detrimental effect, especially for the targeted MiHA-specific T cells relative to the other T cells present in the culture (112). Upon repeated antigen exposure, the MiHA-specific T cells acquired the expression of PD-1 as well as the terminal differentiation marker KLRG-1, which correlated with their relative failure to expand relative to other T cells in the culture. Other research published in the last decade similarly demonstrated that the acquisition of terminal effector T-cell differentiation and exhaustion features *ex vivo*, compromises the further expansion and persistence of the T cells after adoptive transfer. Less-differentiated T cells bearing early memory T-cell features (central memory—Tcm, or stem cell memory—Tscm) have been shown to be superior compared to more differentiated T cells in several animal and human pre-clinical models (113–115). It was also shown in humans that exposure to T-cell memory differentiating factors early in the culture can program long term persistence *in vivo* despite the expression of effector or effector memory T-cell differentiation markers at the end of the culture (102). The issue of T-cell differentiation is relevant to the whole field of T-cell immunotherapy and the quest for culture conditions that will preserve or promote early memory expression is an active area of research. Candidate pathways and molecules shown to influence memory differentiation include cytokines [IL-21 (102, 116), TGF-β (117)] and metabolic/developmental pathways [AKT (118), WNT (119)].

Gene engineering is a way to avoid the drawbacks of using elaborate and long cultures to expand antigen-specific T cells. The transfer of a transgenic TCR in T cells can be achieved using brief manufacturing protocols that maintain early T-cell memory differentiation and that generate a high number of T cells with the desired antigenic specificity. The efficacy and safety of T cells expressing a transgenic HA-1 specific TCR has been established *in vitro* (120). In this study, an elaborate transgene was used for optimal reactivity and safety. The transgene comprised four elements: a TCR specific to HA-1, a CD8 co-receptor to promote the function of the MHC class I restricted TCR in CD4^+^ T cells, an inducible caspase 9 safety switch for rapid induction of apoptosis in case of toxicity and a CD34^−^CD20 tag to facilitate the selection of the cells and to track the cells once transferred (121). This design enabled the expression of the TCR in both CD4^+^ and CD8^+^ cells which may contribute to CD4^+^ T-cell help after transfer. The cells were responsive against different types of primary leukemia cells and cell lines, supporting the further evaluation of HA-1 specific transgenic T cells in clinical trials (NCT03326921). Although the TCR transgenic approach can solve the conundrum of late T-cell differentiation arising in the context of antigen-driven T-cell expansion, it has its own limitations. The production of clinical grade gene therapy vectors is costly and current reports investigating transgenic TCR therapy target only one antigen at a time. TCR transgenic therapies are also limited by the possible mispairing of alpha and beta chain with the endogenous TCR potentially giving rise to unwanted reactivity and toxic allo- or autoimmunity (122, 123). This can be mitigated by the use of murine constant domains, the addition of cysteine residues for preferential pairing of the transgenic chains, α/β chain domain-swapping or the knockdown/out of the endogenous TCR (124–127). However, there is an argument to be made that keeping the endogenous TCR could be beneficial. Chapuis et al. transduced a robust memory EBV-specific T-cell repertoire (which will not cause GVHD) with a TAA-specific TCR transgene in order to leverage the properties of these long term persisting memory cells and use viral reactivations as an adjuvant (103).

Given the possibility for immune escape variants selection following single antigen targeting, the future of MiHA-based therapy may involve multivalent T-cell products (NCT03091933). This emphasizes the importance of discovering and characterizing a large number of MiHA derived from proteins expressed in the hematopoietic system, as well as MiHA presented by enough HLA alleles to treat most, if not all, AHCT patients.

#### TSA

The development of T-cell therapies, or vaccines, against TSA or so-called neoantigens is complicated. Identification and validation of neoantigens is time-consuming as well as expensive. The process of preparing vaccines from tissue samples usually takes several months (62, 63). Finally, the development of TSA-specific T-cell immunotherapy may seem unthinkable given the added complexity of T-cell manufacturing. This being said, several approaches can be taken to leverage TSA identification/prediction and design T-cell immunotherapy. Candidate TSA predicted from mutation analysis have been identified using *in vitro* antigen expression system and co-culture with responder autologous TIL (128). Selection and enrichment of these T-cell populations followed by re-expansion represent an attractive strategy to enhance TIL-based, TSA-specific targeting. Interestingly, circulating T cells recognizing neoantigens detected in cancer patients can be found in the peripheral blood of healthy donors (129). In some cases, the cancer naïve repertoire comprises TSA-specific T cells that are not found in the patient TIL which may indicate the loss of certain T-cell clones in cancer patients. Of particular relevance to the development of T-cell therapies, certain mutations within oncogenes occur at the same genetic location, leading to “public” (or shared) T-cell epitopes (130). An example is the G12D *KRAS* gene mutation in digestive cancers, leading to a mutant peptide presented by HLA-C0802 (131). Such “hot-spot” mutations also exist in blood cancers. A recently published study showed that a frequent nucleophosmin 1 mutation in acute myeloid leukemia resulted in the presentation of a neoepitope by HLA-A0201 (132). Finally, given their “public” nature and restricted expression by cancer cells, the aeTSA may represent excellent targets to investigate for the development of T-cell based immunotherapies of hematological malignancies. However, no human studies have been performed to date with aeTSA.

#### TAA and Viral Antigens

Adoptive T-cell immunotherapy against viral reactivations occurring after AHCT is highly effective, with response rates globally above 70% in otherwise refractory patients (133, 134). In the case of EBV, which is associated with the development of post-transplant lymphoproliferative disorder (PTLD), as well as several lymphoma subtypes outside the context of transplantation, adoptive immunotherapy has a remarkable track record of safety and efficacy (135). Arguably, the prevention or treatment of EBV-associated PTLD after AHCT occurs in the best conditions for T-cell adoptive immunotherapy. The target antigens are foreign, the T cells are expanded (or selected) using multiple antigens from memory T-cell repertoires circulating in immunocompetent healthy donors, who are the original AHCT donors or even partially HLA-matched third-party donors. The resulting T-cell products are polyclonal, can display reactivity against antigens bound by several HLA alleles, usually contain both EBV reactive CD4^+^ and CD8^+^ T cells and, depending on the manufacturing protocol, express early memory T-cell markers. The use of peptide libraries containing multiple epitopes derived from several antigenic EBV proteins (such as LMP2, EBNA1, and BZLF1) allow the generation of multivalent T-cell products (136). Virus-specific T-cell lines are effective after AHCT or even solid organ transplant and can be used as prophylaxis in patients at high risk of PTLD with excellent result and no significant GVHD or organ rejection (137–139). The mobilization of the autologous EBV T-cell repertoire in previously treated lymphopenic lymphoma patients outside the context of transplantation requires more elaborate *ex vivo* culture protocols, but is nonetheless feasible and well-tolerated (107). Bollard et al. reported on 29 patients with EBV-associated lymphoma who received the T-cell lines as consolidation following the achievement of remission (one relapse after a median follow up of 3.1 years) and 21 patients who had active disease at the time of infusion. Among these, 13 had clinical responses (11 complete responses) with evidence of T-cell reactivity against the targeted EBV antigens (LMP1, LMP2) and TAA, evoking the possibility of epitope spreading.

Expanding on the success of anti-viral therapy, it was shown that T-cell lines can be generated by stimulating with overlapping peptide libraries of multiple TAA (140). These T-cell lines products were reactive to multiple TAA simultaneously, were polyclonal, displayed early memory T-cell markers and could be generated from both healthy donors and lymphoma patients. Trials are currently testing the clinical effects of such multivalent TAA-targeting T-cell lines in several blood cancer types (NCT02203903, NCT02494167, NCT02475707, NCT02291848, NCT01333046). Because TAA are molecularly defined and non-polymorphic, they are more easily amendable to transgenic TCR therapy. The isolation and cloning of TAA specific TCR restricted by common HLA alleles can yield TCR sequences that can be used in a large population of patients. For the same reasons, TAA have been used in vaccine trials in the setting of various blood cancers including multiple myeloma, lymphoma, and acute myeloid leukemia [reviewed in Avigan and Rosenblatt (141)]. Transgenic TCR therapy against TAA expressed by hematopoietic cancers was also tested in several clinical trials. Transgenic MHC class I restricted TCR against NY-ESO-1/LAGE-1 and MAGE-A3 have been used to treat myeloma patients. In both cases, the TCR were engineered for increased affinity for the MHC-peptide complex as a way to circumvent a limitation of TAA-based immunotherapy as described above. The use of autologous engineered NY-ESO-1 specific T cells administered in the context of autologous transplantation resulted in clinical responses in 16/20 patients (106). The adoptively transferred T cells showed expansion as well as trafficking to the bone marrow, and did not cause significant toxicity. Expectedly, loss of antigen or lack of persistence of the transferred T cells were associated with relapse. In the case of MAGE-A3, enhanced affinity TCR transgenic T cells caused unexpected and rapid cardiotoxicity in the first 2 patients recruited on the trial (105). Cross-reactivity with a peptide derived from the heart muscle protein TITIN was the causative mechanism. These trials showed both the promise and perils of using affinity enhanced TCR in cancer adoptive immunotherapy. Native and unaltered TAA-specific TCR gene transfer has also been performed. The transcription factor WT1 is overexpressed in several blood cancers and contributes to several known MHC class I associated epitopes. A first trial involving transgenic WT1 specific TCR has been reported in 2017. The study was performed in patients suffering from refractory acute myelogenous leukemia and high risk myelodysplastic syndromes (104). The treatment involved the administration of two T-cell infusions and post-transfer WT1 vaccination. Eight patients were treated in two dose groups. Two objective, but transient, responses were noted and among the five patients who had persisting circulating engineered T cells, four survived more than 12 months. No significant toxicity was observed. More recently, another study was reported using a different transgenic native (but selected for high affinity) TCR against an HLA-A0201 restricted WT1 peptide and transduced in EBV-specific memory T cells (103). The cells were administered to prevent acute myeloid leukemia relapse after AHCT, when the disease burden is low. With a relapse free survival of 100% at a median of 44 months of follow up (compared to 54% in a concurrent control group), an argument can be made about the importance of administering T-cell therapy early in the treatment trajectory of patients.

## Perspectives and Clinical Integration of T-Cell Therapies

The opportunities for antigen-specific T-cell immunotherapies are rapidly expanding. The MHC-associated antigens arising from genetic variants, both germline and acquired through the neoplastic process, are prime targets for the treatment of hematological cancers. The genuinely personalized approaches required to translate the complexity and multiplicity of MiHA, TSA, and TAA into therapy is certainly a challenge, but also a great promise. The discovery and characterization of an increasing number of antigens will enable the design of multivalent therapies capable to target all blood cancers and limit the emergence of immune escape variants associated with single antigen targeting. However, for such promise to materialize, manufacturing processes for these highly personalized therapies will have to be refined and made cost-effective. Nonetheless, T-cell therapies aimed at MHC-associated peptides have the potential to significantly expand existing paradigms in AHCT, autologous cell transfer and other T-cell therapies, such as CAR T cells. Indeed, the development of peptide-MHC specific antibodies may further increase the relevance of characterizing immunogenic MiHA, TAA or shared TSA for CAR-based immunotherapy (142, 143). Along the same lines, genetic variants may also create non-MHC associated cell surface epitopes targetable through recognition by antibodies. Finally, existing CAR may be transduced and expressed in antigen-specific T cells recognizing viral, TAA, MiHA, or TSA through their natural TCR and thus enable dual targeting of malignant cells. The development of multivalent T-cell products, either as a combination of T cells specific for a single antigen or T cells with multiple specificities, will be essential to avoid the emergence of immune escape variants following therapy. In addition, approaches aimed at targeting multiple antigens may prove to be synergistic. For example, pre-clinical studies have shown that only a combination of T cells targeting Y-chromosome derived MiHA and TAA could lead to tumor regression. A threshold effect may be required to generate enough inflammation to support effective anti-cancer immunity (144, 145). Similarly, this is likely achieved in AHCT settings by Y-chromosome antigen-specific T cells given the increased GVT and GVHD effects noted after female into male transplants (146). Another possible benefit of inducing strong immune responses is the development of epitope spreading as evoked by the appearance of detectable anti-TAA responses following microtransplantation, AHCT or anti-viral T-cell therapy (147, 148). Along the same lines, the combination of T-cell therapy with other immunotherapeutic interventions is also likely to unveil important synergies. To this end, the administration of vaccines to consolidate the response after adoptive transfer, or immune checkpoint inhibitor therapy following adoptive T-cell infusion, are actively investigated.

**Figure 3:**
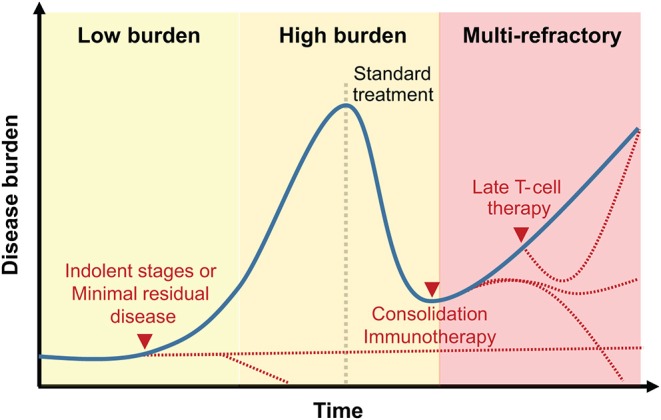
Clinical integration of T-cell therapies targeting MHC-associated antigens. Representation of T-cell therapy timing relative to disease history. While early treatment or treatment following a reduction in disease burden may be associated with prolonged remission (dotted red lines), late-stage blood cancers treatment with MHC-associated antigen-specific T cells may only delay disease progression.

The timing of administration of T-cell therapies will need to be better studied (Figure 3). Cell therapies remain largely offered to refractory patients. However, the promising results following prophylactic DLI (149, 150), anti-viral T-cell lines (137) and more recently transgenic TCR therapy (103), suggest that T-cell therapies should not be confined to the treatment of relapsing patients. In fact, these treatments are probably more potent in the context of low burden disease. The reassuring safety profile of several of the approaches targeting MHC-associated peptides should facilitate the introduction of T-cell therapies earlier on during the patient's course.

Several scientific and methodological issues remain to be addressed to improve T-cell therapies directed against MHC-associated peptides or the MHC molecule itself. A significant contributor to the response against genetic variants is CD4^+^ T-cell mediated, but most identified MiHA, TAA and TSA are MHC class I associated peptides (151). Although challenging, the identification of MHC class II restricted responses will likely be essential to optimize T-cell therapies. This should be a major area of research in the upcoming years.

The downregulation or loss of MHC expression, the genetic loss or silencing of antigen source protein are well-known immune escape mechanisms in cancer. This can be fairly extensive as described in haplo-identical transplants, where the loss of the entire mismatched haplotype can be observed (152). Elaborate strategies targeting MHC-associated peptides presented by different alleles and belonging to different haplotypes may be necessary to harness the therapeutic potential of T-cell immunotherapy against genetic variants translated into MHC-associated peptides. Moreover, an attractive combination approach is to maximize antigen presentation though epigenetic modulation. Demethylating agents, histone deacetylase inhibitors and methyltransferase inhibitors are established or investigational drugs for the treatment of blood cancers. It is increasingly recognized that these also promote gene expression that increases the immunogenicity of malignant cells and also affect immune cell physiology [reviewed in Lindblad et al. (153)]. These effects have been reported to occur through multiple mechanisms like cytokine expression, as well as upregulation of the MHC and associated antigens (154, 155). This last aspect is particularly intriguing as both TAA and cryptic aeTSA antigens have been shown to be promoted by epigenetic modulation (156–159). Notably, extra-exomic endogenous retroviral elements which are attractive as a source of specific and robust cancer antigens can be expressed through modulation of methylation. However, as a note of caution, there is conflicting reports on the outcome of epigenetic modifiers on the physiology of immune cells. Among others, regulatory T cells and the expression of immune checkpoints can be promoted by these agents, perhaps inviting for further combinations with immune modulators.

To conclude, the field is increasingly confronted with multiple antigens and approaches to target them. Careful selection of the best targets will need more research and rational combinations therapies are likely to be required for these antigens to reveal their full potential.

## Author Contributions

All authors listed have made a substantial, direct and intellectual contribution to the work, and approved it for publication. Specifically, VJ, CR, and J-SD contributed to the manuscript design. VJ, CR, SD, CC, and J-SD performed the literature search and contributed to the writing of the manuscript.

### Conflict of Interest

J-SD and CC are authors on patents pertaining to the therapeutic use of minor histocompatibility antigens. The remaining authors declare that the research was conducted in the absence of any commercial or financial relationships that could be construed as a potential conflict of interest.
